# Correlation between an annotation-free embryo scoring system based on deep learning and live birth/neonatal outcomes after single vitrified-warmed blastocyst transfer: a single-centre, large-cohort retrospective study

**DOI:** 10.1007/s10815-022-02562-5

**Published:** 2022-07-26

**Authors:** Satoshi Ueno, Jørgen Berntsen, Motoki Ito, Tadashi Okimura, Keiichi Kato

**Affiliations:** 1 Kato Ladies Clinic, 7-20-3, Nishi-shinjuku, Shinjuku, Tokyo 160-0023 Japan; 2 Vitrolife A/S, Jens Juuls Vej 20, 8260 Viby J, Denmark

**Keywords:** Live birth, Single vitrified-warmed blastocyst transfer, Artificial intelligence, Neonatal outcome, Objective assessment

## Abstract

**Propose:**

Does an annotation-free embryo scoring system based on deep learning and time-lapse sequence images correlate with live birth (LB) and neonatal outcomes?

**Methods:**

Patients who underwent SVBT cycles (3010 cycles, mean age: 39.3 ± 4.0). Scores were calculated using the iDAScore software module in the Vitrolife Technology Hub (Vitrolife, Gothenburg, Sweden). The correlation between iDAScore, LB rates, and total miscarriage (TM), including 1st- and 2nd-trimester miscarriage, was analysed using a trend test and multivariable logistic regression analysis. Furthermore, the correlation between the iDAScore and neonatal outcomes was analysed.

**Results:**

LB rates decreased as iDAScore decreased (*P* < 0.05), and a similar inverse trend was observed for the TM rates. Additionally, multivariate logistic regression analysis showed that iDAScore significantly correlated with increased LB (adjusted odds ratio: 1.811, 95% CI: 1.666–1.976, *P* < 0.05) and decreased TM (adjusted odds ratio: 0.799, 95% CI: 0.706–0.905, *P* < 0.05). There was no significant correlation between iDAScore and neonatal outcomes, including congenital malformations, sex, gestational age, and birth weight. Multivariate logistic regression analysis, which included maternal and paternal age, maternal body mass index, parity, smoking, and presence or absence of caesarean section as confounding factors, revealed no significant difference in any neonatal characteristics.

**Conclusion:**

Automatic embryo scoring using iDAScore correlates with decreased miscarriage and increased LB and has no correlation with neonatal outcomes.

**Supplementary information:**

The online version contains supplementary material available at 10.1007/s10815-022-02562-5.

## Introduction

Single vitrified-warmed blastocyst transfer (SVBT) is increasingly used as the main strategy for in vitro fertilisation (IVF) and embryo transfer treatment in many IVF laboratories [1–3]. SVBT enables the selection of the best blastocyst for transfer and promotes synchronisation and bidirectional communication between a receptive uterus and an implantation-competent blastocyst. Therefore, SVBT prevents multiple conceptions and improves implantation and pregnancy rates as well as neonatal outcomes.

Live birth (LB) is the most important outcome of an IVF treatment. The accurate prediction of LB can reduce the cost and time of IVF by preventing implantation failure and miscarriage. Therefore, assessing embryos by predicting LB is important. In regard to blastocyst assessment, morphological grading based on the Gardner criteria is the most commonly used assessment method [4]. Studies of LB prediction based on blastocyst morphology suggested that the trophectoderm (TE) morphology and blastocyst expansion score can predict live birth [5, 6]. Additionally, determining the inner cell mass (ICM) grade may reduce the risk of early pregnancy loss [7]. As a step further, assessment of embryos by morphokinetic parameters using a time-lapse incubation system was reported as a promising strategy for LB prediction [8–10]. However, blastocyst assessment by Gardner criteria as well as embryo assessment by morphokinetic parameters is subjective and it has been reported that assessments are affected by both intra- and inter-observer variation [11]. Therefore, existing embryo and blastocyst grading systems must be improved by incorporating more objective assessment and reproducible variables.

Recently, the benefit of artificial intelligence (AI) technology for diagnostic purposes in human ART has been shown, particularly for pregnancy prediction after embryo transfer [12–15]. The use of AI for LB prediction after blastocyst transfer has been extensively studied [12, 13, 16–18]. AI can overcome the issue of subjective assessment for selecting blastocysts for transfer. The iDAScore® v1.0 model (Vitrolife, Gothenburg, Sweden) was developed based on the IVY deep learning model [19], and has been integrated directly into a time-lapse system. Therefore, extra equipment or image processing in another computational platform is not required and the IVF laboratory has direct access to embryo scores through the installed software. Our previous study suggested that objective embryo assessment using iDAScore performs as good or even better than traditional embryo assessment or more annotation-dependent ranking tools in various maternal age groups [20]. Furthermore, iDAScore does not require any manual, user-dependent annotations, enabling consistent and objective assessment of blastocysts.

Previously published studies suggested that blastocyst quality, which was based on manual traditional assessment of developmental speed and blastocyst morphology, does influence neonatal outcomes [21–25]. On the other hand, the correlation between basic neonatal outcomes and scores that are calculated by any automatic, AI-based blastocyst scoring system has not been evaluated yet and this is also true for iDAScore. Therefore, the clinical application of iDAScore still requires studies that analyse its correlation with neonatal outcomes.

The objective of this retrospective large cohort study was to investigate whether iDAScore correlates with LB and neonatal outcomes after autologous SVBT.

## Material and methods

### Patients and study design

A total of 3010 patients undergoing 3010 autologous SVBT cycles (1 patient: 1 cycle) at the centre was included between January 2019 and May 2020. In this study, preimplantation genetic testing (PGT) was not involved. During the study period, only single-embryo transfers were performed in our centre, and an exclusive single-embryo transfer policy was strictly followed. No pre-implantation genetic testing was performed. LB and neonatal outcomes were ascertained for all patients using information from a written patient questionnaire and/or from the treating obstetrician. Patients without a complete time-lapse sequence, e.g. due to instrument maintenance, were excluded from the study. Furthermore, iDAScore can only be calculated for embryos that are cultured for at least 112 h after insemination. Therefore, cycles where this time period was not reached were excluded. SVBT was performed on days 4.5–5 after ovulation during a spontaneous natural cycle. The survival rate after thawing was 99.9% (3010/3012). The cycles were stratified into five maternal age groups described by the Society for Assisted Reproductive Technology (< 35, 35–37, 38–40, 41–42, and > 42 years old).

All transferred embryos were evaluated retrospectively using iDAScore v1.0 (Vitrolife) as an annotation-free scoring system based on deep learning. The iDAScore groups were divided into quartiles (1.0–7.2, 7.3–8.6, 8.7–9.2, and 9.3–9.9).

The main outcome measure was the LB rate (LB at ≥ 22 weeks of pregnancy) per embryo transfer procedure. Secondary outcomes were total miscarriage and basic neonatal outcomes, which were analysed for 752 singleton deliveries.

### Minimal ovarian stimulation, oocyte retrieval, fertilisation procedures, and embryo culture

All patients underwent the minimal ovarian stimulation protocol described in previous studies [26]. Intracytoplasmic sperm injection (ICSI) was performed at 4 to 5 h after egg retrieval (ER). Following ICSI, oocytes were transferred to a pre-equilibrated EmbryoSlide (Vitrolife) and incubated in a time-lapse incubator (EmbryoScope^+^ or EmbryoScope Flex, Vitrolife). Embryo slides were prepared according to the manufacturer’s instructions. A one-step medium (NAKA Medical, Tokyo, Japan) was used for embryo culture. The culture dishes were covered with mineral oil (Ovoil, Vitrolife), and all embryos were cultured at 37 °C under a gas phase of 6% CO_2_, 5% O_2_, and 89% N_2_ from day 1 to day 5, 6, or 7.

### Embryo observation, blastocyst monitoring, and vitrification

Normally fertilised zygotes with two pronuclei were cultured until the blastocyst stage. Embryos were observed using EmbryoViewer software without removing the culture dish from the incubator. For vitrification on day 5 or 6, blastocysts were required to attain an inner diameter of > 160 μm [27]. These blastocysts were vitrified immediately according to the Cryotop method [28]. If the developing embryo did not fulfil the criteria, it was cultured for a maximum of 7 days. For blastocyst vitrification on day 7, blastocysts were required to attain an inner diameter of > 180 μm [29]. If an embryo did not fulfil these criteria by day 7, it was discarded. The blastocyst inner diameter was measured using EmbryoViewer software.

### Deep learning model (iDAScore)

The iDAScore v1.0 model [30] was developed using a 3D convolutional neural network and was trained on time-lapse sequence images [19]. The only input to the model was the images of a time-lapse sequence, and the output was a numerical score of 1–9.9, correlating with the likelihood of obtaining a positive foetal heartbeat. Therefore, iDAScore used no subjective human-annotated data for training, and thus no morphokinetic variables were required.

The training data for iDAScore included data from our clinic. However, all data in the current study was obtained after the data used for training. The transferred blastocysts were retrospectively scored using the model from the commercially available iDAScore v1.0.

### Post-warming embryo culture and vitrified-warmed blastocyst transfer procedure

The embryo transfer procedure was performed as described previously [2]. In 2076 cycles only one vitrified blastocyst was available for SVBT. In 934 cycles, more than one blastocyst was available and blastocysts were selected for warming using our original grading system [29, 31] and applied prior to freezing. According to our previous study, assisted hatching using a laser (Saturn 5, CooperSurgical, USA) was performed after warming [32]. Surviving blastocysts were cultured for 30 min to 2 h until blastocoel re-expansion was confirmed. Only blastocysts in which the blastocoel size remained the same or increased relative to the size before vitrification were transferred, whereas degenerating blastocysts were discarded.

Luteal support was provided depending on the blood progesterone level on the day of embryo transfer. Patients with a serum progesterone level > 12 ng/mL on the day of embryo transfer were only administered dydrogesterone (30 mg/day orally; Daiichi Sankyo, Tokyo, Japan). SVBT was not performed in patients with serum progesterone levels of < 8 ng/mL. Patients whose progesterone levels were 8–12 ng/mL were administered progesterone intravaginally (Luteum Vaginal Suppository, ASKA Pharmaceutical Co., Ltd., Tokyo, Japan) until the eighth week of pregnancy.

For clinical outcomes, we used the following definitions: chemical abortion, serum hCG level was over 20 IU/m: [33], but no gestational sac; clinical pregnancy, with a confirmed gestational sac at 6–7 weeks of pregnancy; ongoing pregnancy: a confirmed foetal heartbeat at 9 weeks of pregnancy; LB: LB at ≥ 22 weeks of pregnancy; 1st-trimester abortion: gestational sac was confirmed, but no foetal heartbeat; 2nd-trimester abortion: foetal heartbeat was confirmed, but no delivery; and total miscarriage (TM): gestational sac was confirmed, but no delivery.

During the first trimester, pregnancies were followed approximately until 9 weeks of ongoing gestation (confirmed foetal heartbeat), at which point patients were referred to their obstetrician for subsequent care.

### Neonatal outcomes

Neonatal outcomes (excluding monozygotic twins) for gestational age, congenital malformation rate, male sex ratio, birth weight, rate of small for gestational age (SGA), and large for gestational age (LGA) were compared among the iDAScore groups. SGA and LGA in Japan are defined as a foetal growth curve below the 3rd and above the 97th percentile from the registry database of the Japan Society of Obstetrics and Gynaecology, respectively [34]. In addition, correlations between iDAScore and neonatal outcomes were analysed using regression analysis with maternal age, paternal age, maternal body mass index (BMI), parity, smoking, and with or without caesarean section as confounding factors.

### Statistical analysis

A chi-square test with Bonferroni correction was used to compare categorical variables among groups. Nominal variables were analysed using the Cochran-Armitage test to detect trends, and Wilcoxon rank-sum test was used to compare continuous variables. The discrimination performance of the iDAScore model was evaluated using receiver operating characteristic analysis. Multivariate logistic regressions were used to analyse the relationship between iDAScore and LB or TM. Only factors with *P* < 0.1 in univariate logistic regression analysis were included in multivariate logistic regression to calculate the adjusted odds ratios (aORs). Multiple linear regression analysis was used to control impacts by gender and gestational age when analysing association between birth weight and iDAScore. The assumption that the residuals were normally distributed was tested using normal quantile plots. Additionally, *Z* scores were calculated using national birthweight reference [35]. Comparison of AUCs was performed using a paired two-tailed DeLong’s test. An AUC of 0.5 is equivalent to random classification, and 1.0 is equivalent to 100% correct classification. Statistical analyses were performed using JMP software (version 10.0; SAS Institute, Cary, NC, USA) and R (version 3.6.1, 2019–07-05).

## Results

Table 1 shows the participant characteristics for each maternal age group. Paternal age, number of previous egg retrievals and embryo transfer, miscarriage history, and iDAScore significantly differed among the maternal age groups (*P* < 0.05).

**Table 1:** Patient characteristics for each maternal age group

Age (years)		< 35	35–37	38–40	41–42	≥ 43	All
Cycle, *n*		386	514	796	635	679	3,010
Maternal age (± SD)		32.1 ± 2.0a	36.1 ± 0.8b	39.1 ± 0.8c	41.5 ± 0.5d	44.1 ± 1.2e	39.3 ± 4.0
Paternal age (± SD)		35.5 ± 5.0a	38.5 ± 4.4b	41.2 ± 4.7c	43.2 ± 4.6d	45.2 ± 5.1e	41.3 ± 5.7
No. of previous ER (± SE)		2.2 ± 0.2a	2.9 ± 0.2b	3.5 ± 0.2c	5.0 ± 0.2d	7.8 ± 0.2e	4.5 ± 0.1
No. of previous ET (± SE)		1.6 ± 0.2a	2.2 ± 0.1b	2.7 ± 0.1c	3.6 ± 0.1d	4.3 ± 0.1e	3.0 ± 0.1
Miscarriage history (± SE)		0.21 ± 0.03a	0.23 ± 0.03a	0.30 ± 0.02b	0.39 ± 0.03b	0.30 ± 0.03b	0.30 ± 0.01
Aetiology of infertility	Male factor	28.2	27.4	27.3	25.5	26.7	26.9
	Tubal factor	2.6	4.7	4.8	5.5	4.4	4.6
	Endometriosis	4.7	2.5	3.8	2.7	3.0	3.3
	Ovulation disorders	3.6	2.5	1.8	1.4	1.8	2.1
	Uterine or cervical factor	3.6	4.9	4.9	6.5	6.6	5.5
	Combination	15.0	16.5	13.3	11.7	10.3	13.1
	Unknown	42.0	40.9	43.8	46.8	47.1	44.5
	Others	0.3	0.6	0.4	0.0	0.2	0.3
iDAScore (± SD)		8.3 ± 1.4a	8.2 ± 1.5ab	8.2 ± 1.5ab	8.0 ± 1.5b	7.8 ± 1.6c	8.1 ± 1.5

Values are presented as the mean ± SE or %. Different lowercase letters indicate significant differences among maternal age groups (*P* < 0.05)

*ER* egg retrieval, *ET* embryo transfer, *SD* standard deviation, *SE* standard error

**Fig. 1:**
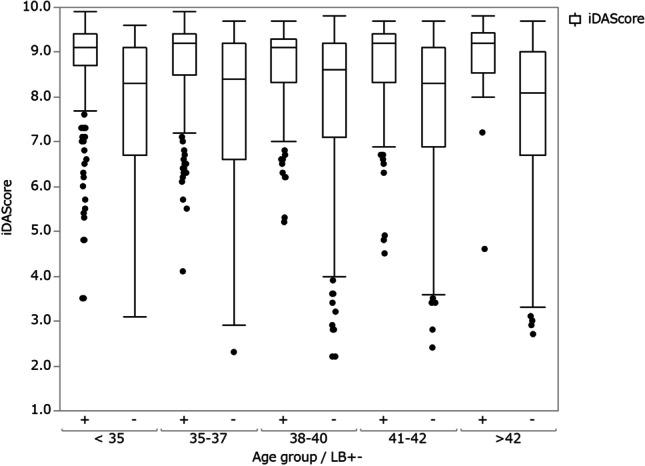
Box plot of distribution of iDAScore for live birth (LB) positive and negative, respectively, within each maternal age group

Table 2 shows the clinical outcomes after SVBT stratified by iDAScore group. For βhCG positive, clinical pregnancy, 1st-trimester miscarriage, ongoing pregnancy, TM, and LB, there was a significant correlation with the iDAScore group (*P* < 0.05). However, no correlation was found between the iDAScore group and chemical abortion and 2nd-trimester miscarriage. Figure 1 shows box plots for the distribution of the iDAScore for LB-positive and LB-negative blastocysts within each age group. Within each maternal age group, iDAScore was significantly higher in the LB-positive group than in the negative group (*P* < 0.05). Furthermore, iDAScores in 42-year-old groups were significantly lower than in other age groups (*P* < 0.05).

**Table 2:** Clinical outcomes after SVBT stratified by iDAScore groups

iDAScore group	9.9–9.3	9.2–8.7	8.6–7.3	7.2–1.0	*P* value
No. of transferred blastocysts	701	798	737	774	-
Maternal age at OR	38.4 ± 4.0^a^	39.0 ± 4.0^b^	39.9 ± 3.8^c^	39.9 ± 3.9^c^	-
βhCG positive (/SVBT, %) *	64.1%	50.3%	34.1%	18.4%	< 0.05
Chemical abortion (/βhCG positive, %)	9.1%	13.5%	13.9%	14.1%	0.12
Clinical pregnancy rate (/SVBT, %) *	58.2%	43.5%	29.3%	15.8%	< 0.05
1st-trimester miscarriage rate (/CPR, %) *	13.7%	12.1%	19.4%	24.6%	< 0.05
Ongoing pregnancy (/SVBT, %) *	50.2%	38.2%	23.6%	11.9%	< 0.05
2nd-trimester miscarriage (/FHB, %)	14.2%	17.1%	20.7%	21.7%	0.17
Total miscarriage* (/CPR, %)	26.0%	27.1%	36.1%	41.0%	< 0.05
Live birth rate (/SVBT, %) *	43.1%	31.7%	18.7%	9.3%	< 0.05
Singleton (/live birth)	99.0%	99.2%	98.5%	100.0%	-
Monozygotic twin (/live birth)	1.0%	0.8%	1.5%	0.0%	-

^*^Significant correlation between the rates of each value and score group (*P* < 0.05)

### LB and miscarriage rates on iDAScore stratified by maternal age

**Fig. 2:**
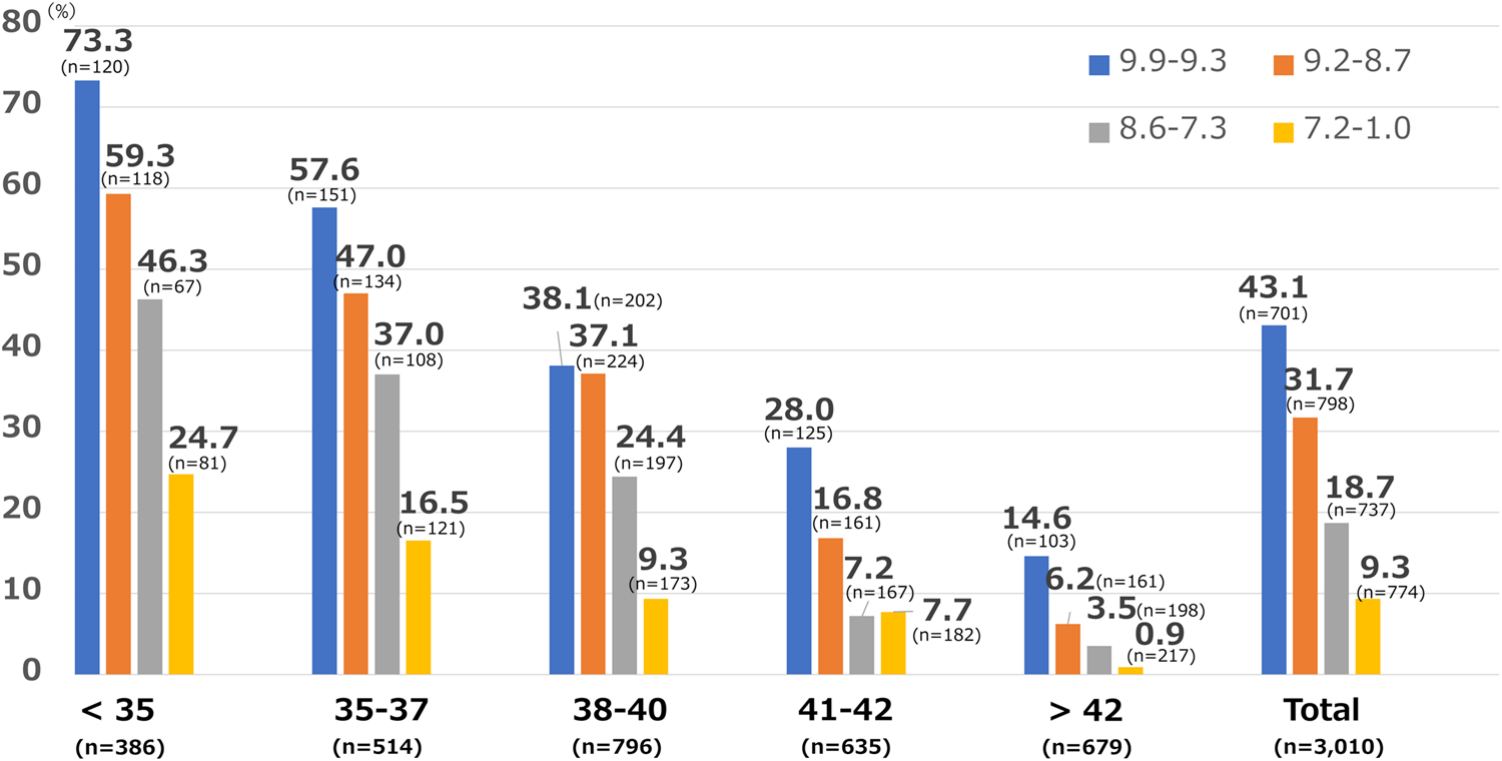
LB rates (%) on the *y*-axis and “Age group” on the *x*-axis. LB rates (%) in subgroups stratified by maternal age group and iDAScore group stratified by quartiles. In each maternal age group, LB rates significantly decreased when the iDAScore group decreased (*P* < 0.05). LB = live birth

We analysed the correlation between LB rates and each iDAScore group stratified by maternal age. The LB rates were significantly different (*P* < 0.05) between groups and decreased progressively with decreasing scores for all maternal age groups (Fig. 2). Table 3 shows the results of univariate and multivariate logistic regression analyses for the LB probabilities. Maternal age, number of previous ER, and iDAScore were significantly correlated with a positive LB probability (maternal age: aOR 0.814, 95% confidence interval (CI) 0.789–0.839; no. of previous ER: aOR 0.963, 95% CI 0.925–0.999; iDAScore: aOR: 1.535, 95% CI: 1.358–1.736, *P* < 0.05). The ability of iDAScore to discriminate between positive and negative LB was estimated using the area under the receiver operating characteristic curve (AUC) metric. The AUC of all patients was 0.700. In the subgroups, the AUCs were as follows: < 35-year age group: 0.705, 35–37-year age group: 0.681, 38–40-year age group: 0.654, 41–42-year age group: 0.694 and ≥ 43-year age group: 0.771.

**Table 3:** Uni- and multi-variate logistic regression analysis for live birth

	Univariate analysis			Multivariate analysis		
	OR ratio	95% CI	*P* value	aOR ratio	95% CI	*P* value
Maternal age	0.797	0.779–0.816	< 0.05	0.814	0.789–0.839	< 0.05
Paternal age	0.906	0.892–0.921	< 0.05	-		0.848
No. of previous ER	0.884	0.861–0.907	< 0.05	0.963	0.926–0.999	< 0.05
No. of previous ET	0.878	0.849–0.907	< 0.05	-		0.775
Cause of infertility	-		0.655	-		
History of miscarriage	0.825	0.717–0.942	< 0.05	-		0.080
Endometrial thickness at SVBT	1.126	1.079–1.174	< 0.05	-		0.178
Luteal phase support protocol (dydrogesterone to luteum)	1.730	0.843–3.570	0.135	-		
Day of vitrified blastocyst						
Day 5	Reference			Reference		
Day 6	0.249	0.198–0.312	< 0.0005	0.638	0.452–0.901	< 0.05
Day 7	0.052	0.007–0.377	< 0.05	0.223	0.028–1.790	0.158
iDAScore	1.811	1.666–1.976	< 0.05	1.535	1.358–1.736	< 0.05

*ER* egg retrievals, *ET* embryo transfers, *OR* odds ratio, *aOR* adjusted odds ratio, *CI* confidence interval, *SVBT* single vitrified-warmed blastocyst transfer

**Fig. 3:**
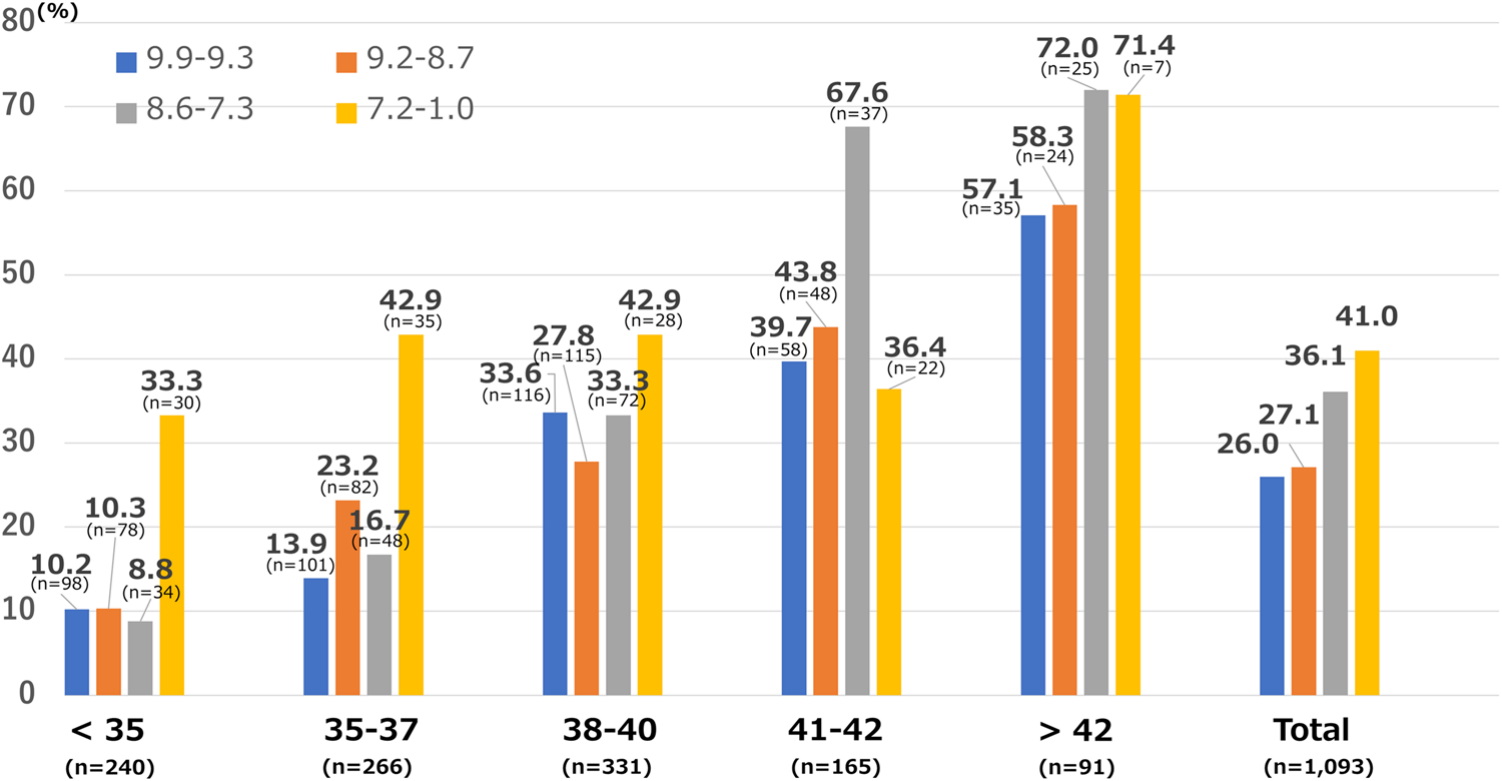
TM rates (%) on the *y*-axis and “Age group” on the *x*-axis. TM rates (%) in subgroups stratified by maternal age group and iDAScore group, stratified by quartiles. TM rates significantly increased progressively with decreasing iDAScores, except in the 38–40-year-old group and > 42-year-old group (*P* < 0.05). TM = total miscarriage

Furthermore, we analysed the correlation between TM and iDAScore groups within each maternal age group. The TM rates significantly increased progressively with decreasing iDAScores, except in the 38–40-year-old group and > 42-year-old group (Fig. 3). Table 4 shows the results of univariate and multivariate logistic regression analyses for TM. Maternal age and iDAScores significantly correlated with TM (maternal age: aOR 1.207, 95% CI 1.154–1.261; iDAScore: aOR: 0.817, 95% CI: 0.716–0.932, *P* < 0.05).

**Table 4:** Uni- and multi-variate logistic regression analysis for total miscarriage

	Univariate analysis			Multivariate analysis		
	OR ratio	95% CI	*P* value	aOR ratio	95% CI	*P* value
Maternal age	1.229	1.180–1.282	< 0.05	1.207	1.154–1.261	< 0.05
Paternal age	1.070	1.045–1.096	< 0.05	-		0.989
No. of previous ER	1.062	1.026–1.100	< 0.05	-		0.679
No. of previous ET	1.060	1.009–1.114	< 0.05	-		0.739
Cause of infertility	-		0.854	-		
History of miscarriage	-		0.133	-		
Endometrial thickness at SVBT	0.891	0.830–0.956	< 0.05	-		0.080
Luteal phase support protocol (dydrogesterone to luteum)	0.403	0.162–1.004	0.051	-		0.127
iDAScore	0.812	0.724–0.912	< 0.05	0.799	0.706–0.905	< 0.05

*ER* egg retrieval, *ET* embryo transfer, *OR* odds ratio, *CI* confidence interval

### Neonatal outcomes

Table 5 shows a comparison of neonatal outcomes among the iDAScore groups after SVBT and singleton delivery. There were no significant differences in the congenital malformation rates, male sex ratio in neonates, rate of caesarean section, gestational age, early preterm birth, very early preterm birth, extremely early preterm birth, birth weight, and rates of LGA and SGA infants. Table 6 shows the unadjusted and aORs for low birth weight, SGA, LGA preterm birth, male sex, and major congenital malformations. Multivariate logistic regression analysis, which included maternal and paternal age, maternal body mass index, parity, smoking, and presence or absence of caesarean section as confounding factors, revealed no significant difference in any neonatal characteristics. Multiple linear regression analysis showed no association between iDAScore and birthweight (Table 7).

**Table 5:** Neonatal outcome–stratified iDAScore group

iDAScore group		9.9–9.3	9.2–8.7	8.6–7.3	7.2–1.0	*P* value
No. of transfer		701	798	737	774	-
Live birth rates		43.1	31.7	18.7	9.3	-
Singleton		297	249	135	71	-
OR maternal age		36.5 ± 3.8	36.7 ± 3.7	37.2 ± 3.3	37.0 ± 3.7	N.S
OR paternal age		38.8 ± 8.3	39.2 ± 6. 0	39.6 ± 5.7	39.9 ± 5.4	N.S
SVBT maternal age		36.7 ± 3.8	36.9 ± 3.7	37.3 ± 3.2	37.1 ± 3.6	N.S
Maternal BMI		20.7 ± 2.8	21.1 ± 2.8	20.8 ± 2.8	20.6 ± 2.4	N.S
Parity		1.4 ± 0.5	1.3 ± 0.5	1.4 ± 0.5	1.3 ± 0.5	N.S
Smoking (%)		1.3%	1.6%	1.5%	1.4%	N.S
Congenital malformation		4.0	4.8	5.2	5.6	N.S
Male sex		52.2	50.2	49.6	43.7	N.S
Caesarean section		31.0	32.1	41.5	32.4	N.S
Gestational age (weeks)		39.1 ± 1.8	39.1 ± 1.7	39.0 ± 1.5	39.2 ± 1.3	N.S
Early preterm birth (32 to < 37 weeks)		7.1	9.2	8.9	8.5	N.S
Very early preterm birth (28 to < 32 weeks)		1.0	0.4	0.0	0.0	N.S
Extremely early preterm birth (< 28 weeks)		0.3	0.0	0.0	0.0	N.S
Birth weight (g)		3017 ± 407	3048 ± 445	3039 ± 397	2996 ± 393	N.S
Male infant	LGA (over 97th P)	1.3	4.8	1.5	0.0	N.S
	SGA (less 3rd P)	0.0	0.8	1.5	0.0	N.S
Female infant	LGA (over 97th P)	2.1	4.8	4.4	0.0	N.S
	SGA (less 3rd P)	0.7	1.6	1.5	2.5	N.S

*BMI* body mass index, *ER* egg retrievals, *SVBT* single vitrified-warmed blastocyst transfer, *LGA* large for gestational age (> 97th percentile), *SGA* small for gestational age (< 3rd percentile), *N.S.* no significant differences in the Cochrane-Armitage trend test

**Table 6:** Unadjusted and adjusted odds ratio in iDAScore for neonatal characteristics in singletons following SVBT

	Unadjusted			Adjusted		
	Odds	95% CI	*P* value	aOdds	95% CI	*P* value
Low birth weight	0.889	0.701–1.166	0.374	1.235	0.691–2.749	0.516
Small for gestation	0.789	0.477–1.631	0.462	2.608	0.361–281.1	0.426
Large for gestation	1.275	0.800–2.446	0.346	0.848	0.454–1.982	0.647
Preterm birth	0.893	0.669–1.264	0.495	0.897	0.420–2.681	0.806
Male sex	1.110	0.961–1.284	0.156	1.166	0.892–1.536	0.267
Major congenital malformation	0.921	0.690–1.305	0.617	0.905	0.487–2.110	0.781

Adjusted for treatment variables were maternal age, paternal age, maternal body mass index, parity, smoking, and with or without caesarean section

**Table 7:** Results of multiple regression analysis of singleton birthweight using the *z* score for birthweight

	Birthweight (g)			*Z* scores		
	*β*	95% CI	*P* value	*β*	95% CI	*P* value
iDAScore	0.009	− 20.680–27.749	0.775	0.009	− 0.062–0.083	0.773
Male sex (versus female)	0.189	54.614–103.21	< 0.05	0.062	0.004–0.149	< 0.05
Gestational age	0.568	127.991–157.254	< 0.05	0.572	0.380–0.467	< 0.05

All variables in the first column were used as confounders for regression analysis

## Discussion

Most of the current embryo selection models based on AI and time lapse have been developed and tested with foetal heartbeat as end point [36]. However, a more clinically relevant end point is live birth. Our study is to our knowledge the largest study where an embryo selection model is tested on SVBT using LB as end point. Few studies have investigated the correlation between embryo selection models and LB predictions [17, 37]. In addition, these models were developed on data from one clinic and, thus, testing of these models did use internal validation data. In contrast, this study can be considered as a temporal external validation as the data was obtained after the development of the model [38]. We identified a correlation between an automatic-embryo evaluation system based on AI (iDAScore) and LB and/or neonatal outcomes using an independent dataset. The ability of iDAScore to discriminate between positive and negative LB within different maternal age groups was lowest for the maternal age group 38–40 and increased toward both younger and older maternal ages. Figure 1 shows that the score distribution of the LB + embryos was quite similar for all age groups. However, mean scores for the LB − embryos were highest for the 38–40 age group and tended to be lower for the younger and older maternal age groups. Thus, it is hypothesised that the difference in discrimination performance is due to that the available useable blastocyst were more homogeneous for the 38–40 age group. For the other age groups, the quality of the available useable blastocysts was more heterogenous and thereby gave rise to a higher AUC due to the easier discrimination.

Currently, embryos are selected for transfer based on our in-house grading system, which grades embryos according to a combination of maternal age and morphology [31]. In order to compare LB prediction performance between iDAScore and our in-house grading system, we adjusted iDAScore for maternal age. The AUC for our in-house grading system was 0.757. For iDAScore, the age-adjusted AUC of 0.794 was significantly higher than in-house grading (Supplemental Table 1). However, it should be noted that when retrospectively comparing AI models against current embryo selection practice there will be an inevitable selection bias [35].

In addition, we found that iDAScore correlated with TM with a significant decrease in TM with increasing iDAScore. A previous study suggested that miscarriage could be predicted based on the trophectoderm grade and embryo morphokinetic [10, 39]. However, evaluation of trophectoderm and direct cleavage is subjective and affected by both inter- and intra-observer variations [40]. A potentially more consistent and generalizable estimation of TM can thus be obtained by using iDAScore. However, due to the nature of deep learning, we currently do not know which features the AI learned. It should be noted that iDAScore has been trained to predict foetal heartbeat (FHB), so the observed correlations with LB and TM must also be important for the likelihood of implantation. This could in example be the genetic or metabolomic competence of the embryo. Further studies are necessary to investigate which input features are important for the AI and miscarriage prediction.

iDAScore did not correlate with basic neonatal outcomes such as malformation, birth weight, gestational age, and male sex rates. Previous studies suggested that blastocyst morphology, including the inner cell mass and trophectoderm, and morphokinetics correlate with sex [21, 41], birthweight [42, 43], and congenital anomalies [44]. However, the correlation between blastocyst quality and neonatal outcomes remains controversial. Several of the studies suggested that blastocyst morphology was related to sex ratio and birthweight [21, 25]. Both studies relied on operator-dependent grading of blastocyst morphology at a single time-point. In contrast, our results were obtained by a completely objective assessment method using timelapse image sequences over the entire embryo development. This study showed no correlation to sex ratio and birthweight, although we observed a non-significant trend of increasing male sex ratio with increasing iDAScores. As pointed out by Afnan et al. [45], it is important to check for unwanted side effects introduced by embryo selection based on black-box AI models. Potential side effects can occur if traits are indirectly correlated with high likelihood of implantation. For example, embryo developmental speed, morphokinetic parameters, and blastocyst grade [21, 40, 46, 47] have been reported to correlate with both sex ratio and implantation likelihood. In a study on iDAScore, it was shown that scores correlate with both time to blastocyst and blastocyst grades [30]. This could possibly be one of the causes for the observed non-significant trend in the male sex ratio. However, such a bias in male sex ratio is also present in current selection methods based on Gardner grading [21, 47]. To get a better estimation of any potential bias in sex ratios, we suggest that AI models should be tested on PGT-A data. This will allow for an estimation of how frequent a male embryo is chosen over a female embryo in a specific cycle. In general, we suggest that black-box models should always be tested for potential unwanted biases and long-term effects.

Our results show that LB probabilities significantly correlate with iDAScore, number of previous ER, day of blastocyst vitrification (day 5 to day 6), and maternal age. Thus, the inclusion of age as input to iDAScore will inevitably improve the overall ranking performance (i.e. the AUC). However, this represents an overoptimistic estimate of ranking performance as age does not improve sorting capability on treatment level where age is constant. Hence, when age is used as model input [16], it is important to test the ranking performance on age sub-groups to get a better understanding of the performance on treatment level. If the aim of an AI model or other blastocyst assessment model are to predict the actual implantation potential in a clinic, it is necessary to make a clinic-specific calibration of the model output using maternal age and other clinic specific covariates [13]. As shown in our previous study, it is possible to make a clinic-specific calibration of KIDScore D5 to reflect the actual chance of an FHB or LB within different age groups [9]. This can be used for patient counselling on whether to transfer a low score blastocyst. In addition, a calibrated model will also allow for a clinic-specific threshold to be used for deciding whether a given blastocyst should be vitrified. It should be noted that a clinic-specific calibration will not change the ranking and transfer order within a single cycle. The number of previous ER correlated with the LB probability. This was probably due to the poor prognosis for patients with many previous ER. The day of blastocyst vitrification also correlated with the LB probability. This suggests that for the prediction of the actual LB probability, iDAScore needs to be calibrated differently for day 5 and day 6. However, a previous study in our clinic suggested that day of blastocyst vitrification did not influence the FHB prediction ability of iDAScore [20]. Therefore, before using iDAScore for LB prediction, clinics should verify if the model should be calibrated differently for days 5 and 6.

The iDAScore model was developed based on the IVY model [19]. The performance of the IVY model was evaluated on a significantly different sub-set of embryos as their test data set regarded discarded embryos as FHB − . Thus, their reported high AUC of 0.93 is a measure of the discrimination between FHB + vs (FHB − and discarded embryos). This is different from the current study where the test data set only includes transferred embryos and specifically discarded embryos are not included in the AUC calculation. In the analysis of iDAScore v1.0 by Berntsen et al. [30], the AUC for FHB + vs (FHB − and discarded) was 0.95 and for FHB + vs FHB − the AUC was 0.67. The differences between these two AUC values clearly show that it is much easier to discriminate between FHB + and discarded embryos than to discriminate among transferred embryos alone. However, from a usability point of view, it is important that an embryo selection model can identify the discarded embryos so that the model can be used on all embryos without any preselection by the user. As described in Berntsen et al. [30], this can be achieved by including discarded embryos in the training data. However, from a clinical perspective, we believe that the most important performance measure is the discriminative performance on a test set with only transferred embryos.

The iDAScore does not always correlate with the results of blastocyst assessment by other models e.g. Gardner criteria or KIDScore D5 [20]. This is one of the issues in embryo assessment by artificial intelligence. To address this issue, the final decision should be done by humans who are medical doctors or embryologists. In this regard, the medical staff who selects embryos for transfer needs to deeply understand embryo assessments models including traditional models. Furthermore, for the time being we recommend that blastocysts should be assessed by morphology or morphokinetics along with AI models. Such data will be helpful in the final decision which blastocyst to select for transfer and in patients counselling, especially when embryo selection by artificial intelligence scoring system is uninterpretable.

A major limitation of our study is that it was based on minimal stimulation and natural cycle IVF treatment, involving only insemination by ICSI and a freeze-all strategy whereby all transferred blastocysts had previously been vitrified. The vitrification procedure may cause a partial damage to blastocysts, thereby decreasing the overall implantation potential. Therefore, the ability of iDAScore to predict pregnancy outcomes may differ between fresh and frozen blastocyst transfers. Consequently, studies of fresh blastocyst transfers are required to determine the efficacy of the iDAScore in regard to TM, LB, and neonatal outcome. The iDAScore model was developed for FHB + prediction using data from 18 fertility centres that use different IVF procedures [30]; however, most of the data were from cycles with a fresh embryo transfer. Further studies performed in other fertility centres with different settings and practices are required to support the general applicability of this approach. As only minimal stimulation was used, studies using standard controlled ovarian stimulation are required to analyse the efficacy of selection in elective blastocyst transfer situations, preferably by using a randomised controlled trial design. Additionally, iDAScore can only be used in a time-lapse system (EmbryoScope^+^) equipped with the adequate software (Vitrolife Technology Hub). In addition, this study was retrospective in nature and thus may have limitations. Therefore, in future studies, randomised controlled trials are required.

## Conclusions

Objective embryo assessment using a completely automatic and annotation-free model, iDAScore, is correlated with LB and TM. Furthermore, iDAScore was not correlated with any neonatal outcome parameters. iDAScore does not require any manual, user-dependent annotations, enabling objective assessment of embryos that are cultured to the blastocyst stage. Therefore, iDAScore is an optimal method for scoring embryos and prioritising blastocysts for transfer without compromising live birth and neonatal outcomes.

## Supplementary information

Below is the link to the electronic supplementary material.

Supplementary file1 (DOCX 14 KB)
