# Correction to: Correlation between an annotation-free embryo scoring system based on deep learning and live birth/neonatal outcomes after single vitrified-warmed blastocyst transfer: a single-centre, large-cohort retrospective study

**DOI:** 10.1007/s10815-022-02605-x

**Published:** 2022-08-31

**Authors:** Satoshi Ueno, Jørgen Berntsen, Motoki Ito, Tadashi Okimura, Keiichi Kato

**Affiliations:** 1 Kato Ladies Clinic, 7-20-3, Nishi-shinjuku, Shinjuku, Tokyo 160-0023 Japan; 2 Vitrolife A/S, Jens Juuls Vej 20, 8260 Viby J, Denmark

**Correction to: Journal of Assisted Reproduction and Genetics**

10.1007/s10815-022-02562-5

The article Correlation between an annotation-free embryo scoring system based on deep learning and live birth/neonatal outcomes after single vitrified-warmed blastocyst transfer: a single-centre, large-cohort retrospective study, written by Satoshi Ueno, Jørgen Berntsen, Motoki Ito, Tadashi Okimura, Keiichi Kato, was originally published electronically on the publisher’s internet portal on 26 of July 2022 without open access. With the author(s)’ decision to opt for Open Choice the copyright of the article changed on 25 of August 2022 to © The Author(s) 2022 and the article is forthwith distributed under a Creative Commons Attribution 4.0 International License, which permits use, sharing, adaptation, distribution and reproduction in any medium or format, as long as you give appropriate credit to the original author(s) and the source, provide a link to the Creative Commons licence, and indicate if changes were made. The images or other third party material in this article are included in the article’s Creative Commons licence, unless indicated otherwise in a credit line to the material. If material is not included in the article’s Creative Commons licence and your intended use is not permitted by statutory regulation or exceeds the permitted use, you will need to obtain permission directly from the copyright holder. To view a copy of this licence, visit http://creativecommons.org/licenses/by/4.0.

The original article has been corrected.

