# Supplementary material for: Correlation between an annotation-free embryo scoring system based on deep learning and live birth/neonatal outcomes after single vitrified-warmed blastocyst transfer: a single-centre, large-cohort retrospective study
Source: J Assist Reprod Genet. 2022 Jul 26;39(9):2089–99. doi: 10.1007/s10815-022-02562-5 (PMC9475010; doi:10.1007/s10815-022-02562-5)
Supplement: Supplementary file 1 — Supplementary file1 (DOCX 14 KB) [file 10815_2022_2562_MOESM1_ESM.docx]

Supplemental Table 1: Adjusted odds ratio for comparison of live birth prediction following SVBT between iDAScore and in-house grading system. Odds were adjusted for maternal age.

|  | Adjusted | | |
| --- | --- | --- | --- |
|  | aOdds (95%CI) | P value | AUC |
| iDAScore | 1.75 (1.60-1.91) | p < 0.05 | 0.794a |
| In-house grading system | 2.62 (2.38-2.89) | P < 0.05 | 0.757b |

Different lowercase letters indicate significant differences among maternal age groups (P < 0.05).
